# Morphometry and displacement analysis of the upper lips following maxillary full-arch implant-supported fixed prostheses: a 3D morphometric study

**DOI:** 10.1186/s12903-021-01838-z

**Published:** 2021-09-23

**Authors:** Keyi Hao, Jia Luo, Ping Di, Yu Zhang, Ye Lin

**Affiliations:** Department of Implantology, Peking University School and Hospital of Stomatology & National Clinical Research Center for Oral Diseases & National Engineering Laboratory for Digital and Material Technology of Stomatology & Beijing Key Laboratory of Digital Stomatology, 22 Zhongguancun South Avenue, Haidian District, Beijing, 100081 People’s Republic of China

**Keywords:** Stereophotogrammetry, Edentulous maxilla, Full-arch, Fixed prosthesis

## Abstract

**Background:**

With the emergence of three-dimensional (3D) integration technology, analysis of soft tissue displacement and morphological changes after maxillary full-arch implant-supported fixed prostheses can be performed. The aim of this study was to verify the feasibility of the 3D integration method for constructing the relative position of the prostheses and facial soft tissue, evaluate the displacement and morphological variation of the upper lips after maxillary full-arch implant-supported fixed prostheses.

**Methods:**

Twenty-five maxillary edentulous patients were recruited in this study. At the time of final prosthesis delivery, the 3D prostheses data and three 3D facial profiles were integrated. After method validation, the 3D position changes of seven soft tissue landmarks were used to reflect the 25 upper lips. The variation of four morphological distances were analyzed to reflect the morphological alteration of the upper lips. Two pairs of dentofacial landmarks were used to analyze the sagittal relative position of the prostheses and soft tissue. The included patients were also grouped to determine the impact of sex, upper lip thickness, and length on lip support changes.

**Results:**

The average distance of the two matched relative reliable forehead regions was only 0.32 mm. The sagittal shifts of labrale superius (LS), stomion (STO), crista philtri left (CPHL) and crista philtri right (CPHR) were 3.44 ± 1.39 mm, 2.52 ± 1.38 mm, 3.04 ± 1.18 mm, and 3.12 ± 1.21 mm, respectively. With the exception of the decrease in the length of subnasale (SN)-LS, the length of cheilion right (CHR)-cheilion left (CHL), CPHR-CPHL, and LS-STO significantly increased. The two pairs of dentofacial landmarks had strong positive movement correlations along the sagittal direction. Patients with thinner and longer lips showed more lip support than those with thicker and shorter lips by a clinically insignificant amount.

**Conclusions:**

The integration method of 3D facial and dental data showed high repeatability in constructing the dentofacial relative position. The linear equations reflecting dentofacial relative position could aid clinicians in evaluating the restoration effect and estimate the upper lip variation.

## Background

The widely used full-arch fixed implant-supported prostheses provide better outcomes in terms of great stability, survival rates, and less invasiveness [1, 2]. However, the extent to which upper lip support can be provided by this flangeless fixed denture, the changes in the upper lip, the dentofacial sagittal movement relationship, and related influencing factors have not been confirmed by sufficient studies. The position and morphological changes of the upper lip after denture wearing are important aspects for evaluating the effect of maxillary full-arch implant-supported prostheses from an esthetic point of view.

Based on two-dimensional (2D) photographs [3] and cephalometric analysis [4], the vertical height of the upper lip and the upper vermilion show a significant increase following complete removable dental prostheses [5]. Mirjam et al. [6] have reported the relationship between different incisor movements and the soft tissue change using 2D cephalograms. The movement ratio between the most anterior point of the maxillary central incisor and labrale superius (LS) was 1:0.59. However, with respect to maxillomandibular advancement surgery, every millimeter movement of the most prominent point on the maxillary incisor can be followed by a change of 0.96 LS [7].

With the development of digital techniques, digital facial profiles and three-dimensional (3D) data make facial evaluation more objective and accurate. It was reported that after the restoration of complete removable dental prostheses, the average sagittal movement of the upper lip was 7.7 mm [8]. The sagittal shifts of crista philtri left (CPHL) and crista philtri right (CPHR) were 3.65 ± 0.21 mm and 3.81 ± 0.17 mm, respectively [9]. The 3D technique was also applied for upper lip analysis after Le Fort I advancement surgery. The sagittal soft tissue changes at subnasale (SN), LS, and stomion (STO) were 5.2 ± 2.0, 5.8 ± 2.5, and 5.2 ± 1.8 mm, respectively. According to a systematic review, a ratio of 0.6:1 (LS to upper incisor [UI]) could be used after Le Fort I advancement surgery [10]. However, changes in the soft tissue of the upper lip following maxillary full-arch implant-supported fixed dentures have rarely been reported. Although Tartaglia et al. [11] has once pointed out that both the height of the upper lip and the width of the mouth are increased after implant-supported fixed denture wearing, the specific quantitative 3D soft tissue changes and the relative relationships need to be further explored.

The 2D photographs showed the higher combined total error for linear and angle measurements (0.78 mm, 0.35°) than 3D laser scanning (0.50 mm, 0.25°) and stereophotogrammetry (0.28 mm, 0.09°) [12]. Moreover, both the stereophotography and structured light satisfied oral clinic requirements without significant difference in accuracy [13]. 3D technology can not only show the precise quantitative changes of soft tissue in 3D space [14, 15] but also reveal the spatial volume changes of soft tissue in more detail [16], which are unattainable by the original 2D technology.

In 2008, Rangel reported that it was technically possible to obtain a 3D digital face with a visible dental cast [17]. Rosati [18] and Bassam [19] have also demonstrated that merging of the digital dental maxillary arch and 3D facial profile could reproduce dentofacial relationships. These results are essential for subsequent studies and provide an important matching method for 3D data analysis.

Although 3D integration technology is technically possible, it remains unclear whether it can be applied accurately and effectively to analyze dentofacial relationships after implant-supported restorations. Thus, the aims of the study were to verify the feasibility of the 3D integration method for constructing the relative position of the prostheses and facial soft tissue in the same 3D coordinate system, evaluate the displacement and morphological variation of the upper lips following maxillary full-arch implant-supported fixed prostheses, and explore the relationship and the soft tissue related factors between the soft tissue changes and the position of the restoration.

## Methods

### Participant recruitment

All consecutive patients (n = 25, 11 women and 14 men) with a mean age of 59 (range, 41–72) years with edentulous maxillae were included from the Department of Oral Implantology, Peking University School and Hospital of Stomatology. The following inclusion criteria [20, 21] were considered for patient selection: patients (1) with edentulous maxilla without skeletal malocclusion, (2) with natural lower dentition or lower full-arch restoration that was previously completed, and (3) with sufficient maxillary bone to maintain at least four 10-mm implants. The exclusion criteria [22] were as follows: (1) patients with any uncontrolled systemic disease that prevents surgery, (2) patients with clenching or grinding teeth while sleeping, (3) heavy smokers (≥ 20 cigarettes daily), (4) patients with poor oral hygiene or poor compliance, and (5) patients with a history of radiotherapy or bisphosphonate treatment.

This study was approved by the Animal Ethics Committee of Peking University School and Hospital of Stomatology (PKUSSIRB-201839137). The experiments were performed in accordance with the approved guidelines and regulations.

### Three-dimensional (3D) data acquisition

Four to six implants were inserted into the maxillary bone with at least 35 N-cm torque for all selected patients. The temporary denture was delivered on the day of surgery. The final denture was delivered approximately 6 months after the surgery. According to the principle of denture design, the central top point of the acrylic flange border (F) is located 1 mm above the maximum smile line. The UI point is designed to be 2 mm below the lower edge of the upper lip and 10 mm in front of the center of the incisive papilla [8].

At the time of the final prosthesis delivery, Face Scan (Isra Vision, Darmstadt, Germany) was used to capture 3D images of patients. To avoid subtle changes in the soft tissue caused by the patient’s different head position and occlusal position, a laser gradienter was used to make the patient’s Frankfort plane parallel to the horizontal plane, ensuring that the natural head position was achieved [23–25]. During this part, three 3D facial profiles of the same patient were obtained [18]: (1) the profile with the collapsed upper lip in rest position without the final denture, (2) the profile with the teeth in rest position and closed lips after the final denture fixed, and (3) the profile with the cheek retractors and the visible frontal teeth in the intercuspal position. The rest position was achieved upon full relaxation for 1 or 2 s and then by pronouncing the word “Emma” [26]. In addition, the 3D data of the final maxillary denture were captured in the IScan D104i (Imetric 3D SA, Switzerland).

### 3D coordinate system establishment

After importing all 3D data into Geomagic Studio 2013 software (Raindrop Geomagic, United States), a 3D coordinate system was established. The X-axis was established by sequentially connecting the left outer canthus point (OCL) and right outer canthus point (OCR), and the rightward direction was defined as positive. The midpoint of the line connecting the two outer canthus points was set as the coordinate origin. These three points can be used to establish the XZ plane. The forward direction on the Z-axis was set as the positive direction. The Y-axis was perpendicular to the X- and Z-axes and passed through the origin point. The downward direction of the Y-axis was considered positive.

### Data integration and method validation

**Fig. 1:**
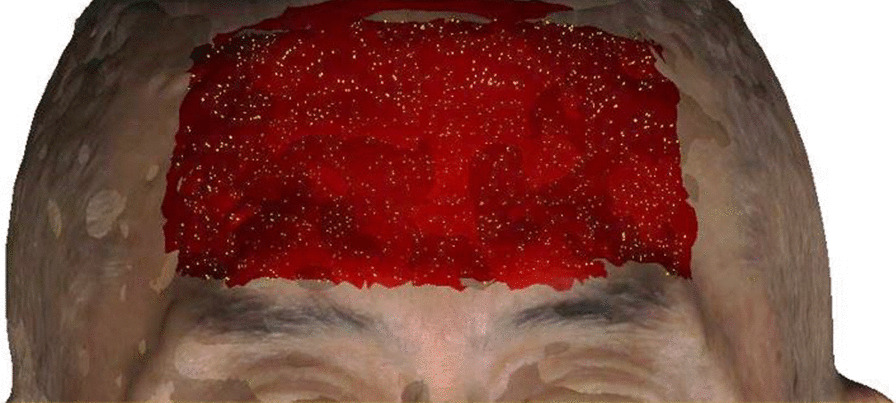
The illustration of the forehead region from the hairline to the upper edge of the eyebrow for the facial data integration

These 3D data were integrated in a common coordinate system. The initial alignment was performed by identifying five corresponding landmarks (inner/outer canthus points, left/right portion, and nasion [N] point) spread over the 3D facial data with closed lips before and after the final denture was worn [17]. The second alignment was performed by selecting the corresponding regions. During this process, the forehead region from the hairline to the upper edge of the eyebrow was selected as a relatively reliable region to complete the integration (Fig. 1). Furthermore, the above matching method was also applied to integrate digital maxillary implant-supported denture and facial images with cheek retractors. However, considering the slight deformation of the lateral parts of the denture, only the upper central and lateral incisors were selected as reliable matching regions.

The mean distance in the selected forehead regions between the profiles with and without the final denture was calculated. The following seven repeatedly located facial and dental landmarks (SN, LS, the central top point of the acrylic flange border [F] [27], UI, N, OCL, OCR) were obtained directly on the 3D digital integrated images of 12 patients [18, 28]. The operator then connected these points and completed the linear measurements. The 3D data of the 12 patients were integrated and measured twice by two experienced doctors.

### Soft tissue change and effect factor analysis

**Fig. 2:**
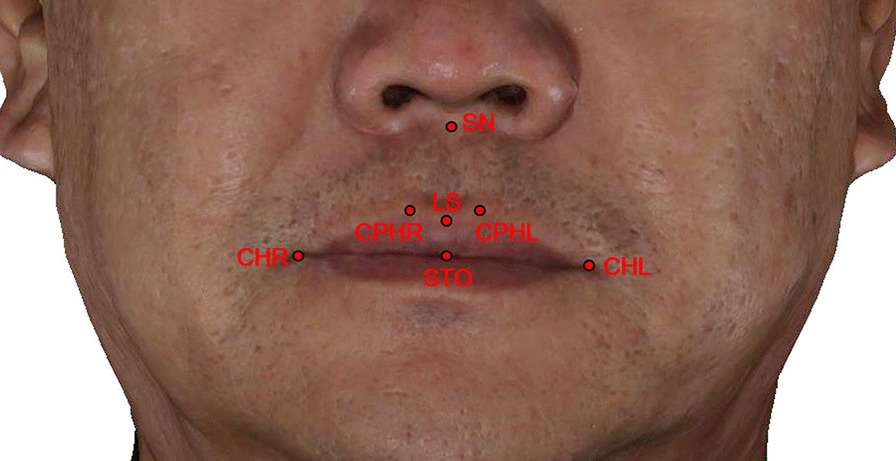
The landmarks of the three-dimensional facial profile for a patient with final prosthesis

**Fig. 3:**
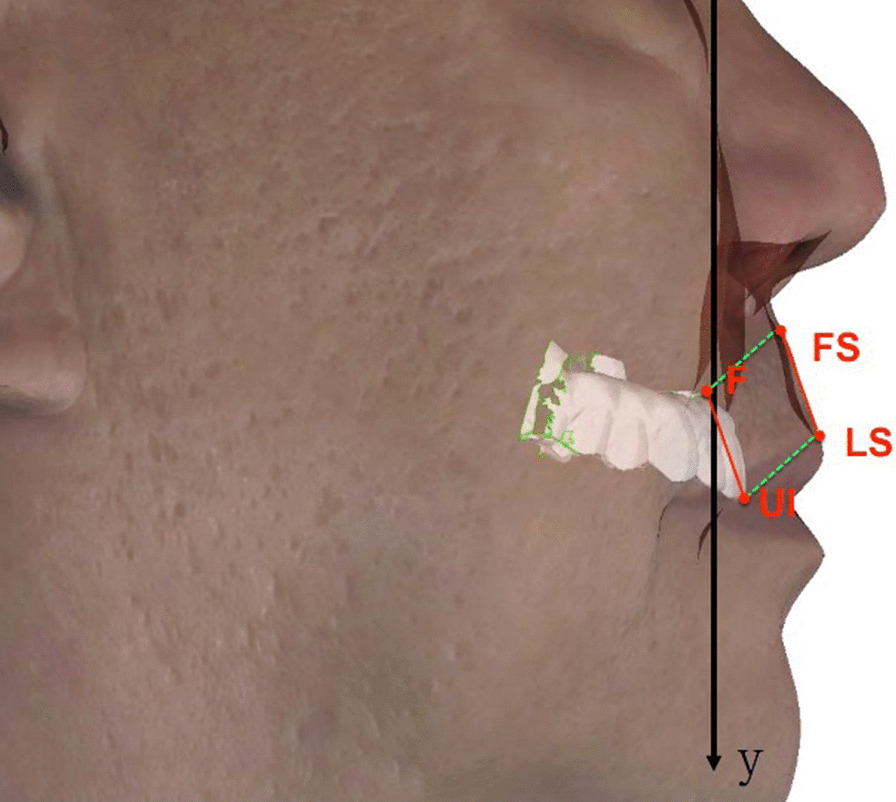
The illustration of the corresponding points between prostheses and upper lips

The following soft tissue and dental landmarks (cheilion left [CHL], cheilion right [CHR], CPHL, CPHR, LS, SN, STO, UI, F, the intersection of the parallel line of UI-LS through point F and the surface of the soft tissue [FS]) were obtained on the surfaces of digital dentures and the upper lips (Figs. 2 and 3). These morphological distances (SN-LS, CPHR-CPHL, CHR-CHL, LS-STO) were also measured to reflect the detailed changes in soft tissue morphometry (Table 1).

**Table 1:** Landmarks and measurements used in the study

	Definition
*Landmarks*	
SN	Subnasale: the point at which the nasal septum merges with the upper lip
LS	Labrale superius: the midpoint of the upper vermilion line
STO	Stomion: the most inferior point on the upper lip
CPHL	Crista philtri left: the point on the left elevated margins of the philtrum above the vermilion line
CPHR	Crista philtri right: the point on the right elevated margins of the philtrum above the vermilion line
CHL	Cheilion left: the point located at the left labial commissure
CHR	Cheilion right: the point located at the right labial commissure
UI	Upper incisor: the tip of the maxillary central incisors
F	The central top point of the acrylic flange border
FS	The intersection of the parallel line of UI-LS through point F and the surface of the soft tissue
OCL	Left outer canthus point: the outermost point at the commissure of left eye's upper and lower eyelids
OCR	Right outer canthus point: the outermost point at the commissure of right eye's upper and lower eyelids
N	Nasion: most anterior point of the frontonasal suture where the lines of the glabella profile meet those of the nasal bones
*Measurements*	
SN-LS	Upper white lip length
CPHL-CPHR	Philtrum width
CHL-CHR	Mouth width
LS-STO	Upper vermillion height
ULT	Upper lip thickness: distance between LS and the most anterior point of the maxillary incisor
ULL	Upper lip length: distance between SN and LS

The upper lip thickness (ULT, distance between the LS and the most anterior point of the maxillary incisor) [29] and upper lip length (ULL) could be directly measured in the 3D coordinate system. In addition to the male and female groups, the 25 patients were also divided into the thin-lip group (ULT < 11 mm, n = 13) and the thick-lip group (ULT ≥ 11 mm, n = 12) according to the Chinese Han national criteria for the thickness of the upper lip [30]. The 25 patients were also divided into the long-lip group (ULL ≥ 20 mm) and the short-lip group (ULL < 20 mm) to analyze the influence of lip support.

### Statistical analysis

The sample size was calculated using R (R Core Team, 2019). Specifically, the power and significance level were set to 0.9 and 0.05 respectively. The required sample size N obtained from the software was 9.

Statistical analysis was performed using the Statistical Package for the Social Sciences (SPSS) Statistics version 19 (SPSS Inc., Chicago, IL, USA). In this study, Wilcoxon signed-rank tests were used to analyze integration reproducibility. *P*-values < 0.05 were considered significant. The mean absolute difference (MAD) and technical error of measurement (TEM) were also calculated to verify the integration accuracy and reliability.

Regarding landmark variation after denture wearing, the Shapiro–Wilk test was used to verify whether the data were normally distributed. Subsequently, the paired t-test was used to analyze the selected distances where the data were normally distributed. The Wilcoxon test was used when data were not normally distributed. The average displacement changes of the landmarks in three different directions were compared using analysis of variance. The independent-samples t-test was used to analyze the lip support difference between patients with different crucial influencing factors, such as lip thickness, lip length, and sex. Linear regression analyses between the sagittal movement of the F and FS and UI and LS were performed using R.

## Results

### Integration method validation

According to the linear measurement of facial and dental landmarks, the integration procedure of 3D facial profiles and digital dentures showed high accuracy. The evaluation of reliability and repetitiveness of the matching method is presented in Table 2. No significant differences were found between the two groups. MAD values were < 0.55 mm, ranging between 0.17 and 0.53 mm. TEM values were also < 0.55 mm, ranging between 0.19 and 0.52 mm. Moreover, as shown in Table 3, the average distance of the two matched relative reliable forehead regions was only 0.32 mm (standard deviation, 0.17 mm). These results reflect the acceptable clinical applications of this matching method.

**Table 2:** The evaluation of integration reproducibility

Distance (mm)	First match		Second match		MAD	CI	TEM	*P*
	Mean (mm)	SD	Mean (mm)	SD				
F-OCR	74.70	3.34	74.45	3.35	0.26	[− 0.14, 0.65]	0.23	0.63
F-N	61.27	4.39	61.02	4.41	0.25	[− 0.28, 0.79]	0.26	0.76
F-OCL	75.35	4.09	75.23	3.85	0.53	[− 0.54, 1.59]	0.52	0.89
UI-OCR	85.30	3.06	85.36	3.19	0.30	[− 0.27, 0.87]	0.29	0.98
UI-N	73.74	3.39	73.79	3.35	0.20	[− 0.15, 0.55]	0.19	0.98
UI-OCL	86.22	3.99	86.31	4.03	0.17	[− 0.27, 0.62]	0.20	0.98
SN-OCR	67.57	2.74	67.69	3.00	0.43	[− 0.12, 0.98]	0.36	0.80
SN-N	50.79	2.64	51.07	2.72	0.49	[− 0.47, 1.45]	0.48	0.80
SN-OCL	69.20	2.91	69.45	3.04	0.47	[− 0.55, 1.50]	0.49	0.84
LS-OCR	82.20	3.19	82.09	3.17	0.38	[− 0.26, 1.02]	0.35	0.89
LS-N	68.80	3.14	68.61	3.07	0.48	[− 0.40, 1.36]	0.46	0.84
LS-OCL	83.83	3.32	83.63	3.08	0.42	[− 0.45, 1.29]	0.42	0.84

*P*-values from the Wilcoxon signed-rank test (*P* < 0.05)

**Table 3:** The average distance of the two matched surfaces in the forehead regions

Subject	Average distance (mm)	SD
1	0.16	0.11
2	0.24	0.17
3	0.32	0.20
4	0.15	0.14
5	0.21	0.13
6	0.58	0.23
7	0.26	0.15
8	0.31	0.18
9	0.47	0.22
10	0.29	0.19
11	0.49	0.12
12	0.39	0.19
Mean	0.32	0.17

### Morphological variation of the upper lip

**Fig. 4:**
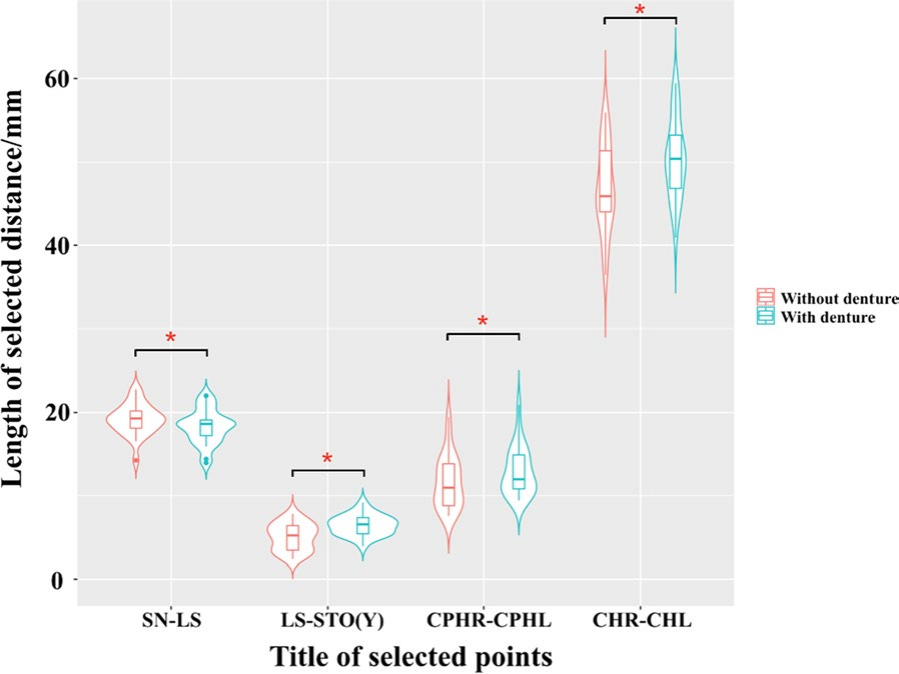
The violin plot shows the variation of distance before and after the fixed restoration of maxillary implant-supported prostheses; LS-STO(Y) represents the vertical distance between the two points; *P < 0.05

The violin plot shows the distribution and variation of the morphological distance between the landmarks before and after the fixed restoration of maxillary implant-supported prostheses. With the exception of the decrease in the length of SN-LS (0.88 ± 0.94 mm), the length of CHR-CHL (3.50 ± 1.84 mm), CPHR-CPHL (1.22 ± 0.68 mm), and LS-STO (1.41 ± 0.75 mm) significantly increased (*P* < 0.05) (Fig. 4).

**Fig. 5:**
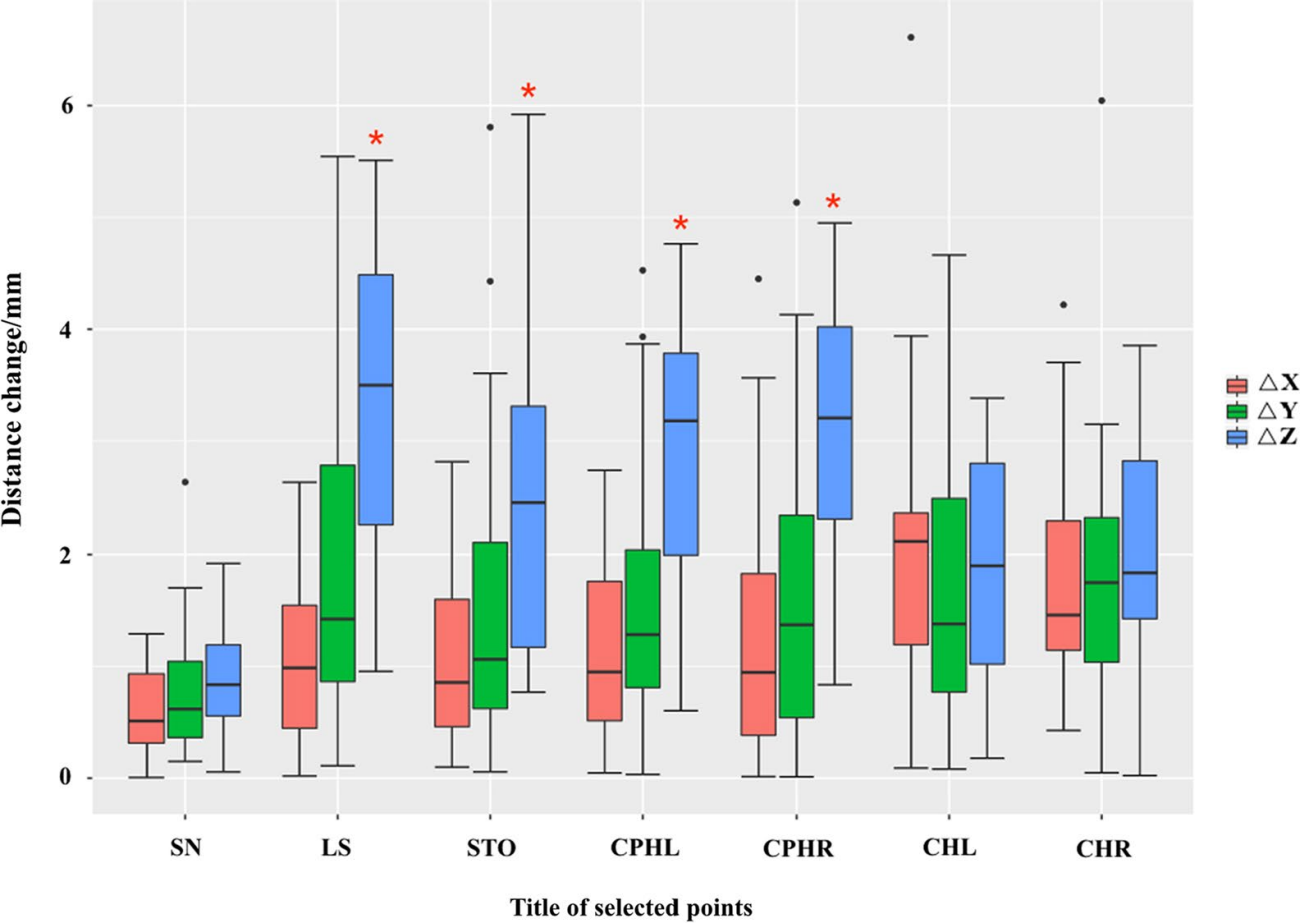
Comparison of average displacement changes of landmarks in different directions before and after the restoration (△ = change), *P < 0.05

Regarding the displacement after wearing the final full-arch maxillary implant-supported prostheses, all the landmarks on the soft tissue moved forward. The nasal base area changed minimally, and the shift of SN in the sagittal direction was only 0.61 ± 0.44 mm. However, the sagittal shifts of LS, STO, CPHL, CPHR, CHL, and CHR were 3.44 ± 1.39, 2.52 ± 1.38, 3.04 ± 1.18, 3.12 ± 1.21, 1.94 ± 1.01, and 2.01 ± 1.01 mm, respectively. In the vertical direction, the SN, LS, CPHL, and CPHR moved upward, and their vertical shifts were 0.79 ± 0.59, 1.891.51, 1.661.21, and 1.651.32 mm, respectively. However, STO, CHL, and CHR slightly moved downward, and their vertical shifts were 1.57 ± 1.41, 1.71 ± 1.24, and 1.76 ± 1.22 mm, respectively. This change demonstrated that the dentures extended the height of the soft tissue by supporting and stretching them. Considering the different directions, LS, STO, CPHL, and CPHR, located in the middle part of the upper lips, significantly moved forward along the Z-axis than moved in the direction of the X- and Y-axes (Fig. 5).

### Linear regression analysis

**Fig. 6:**
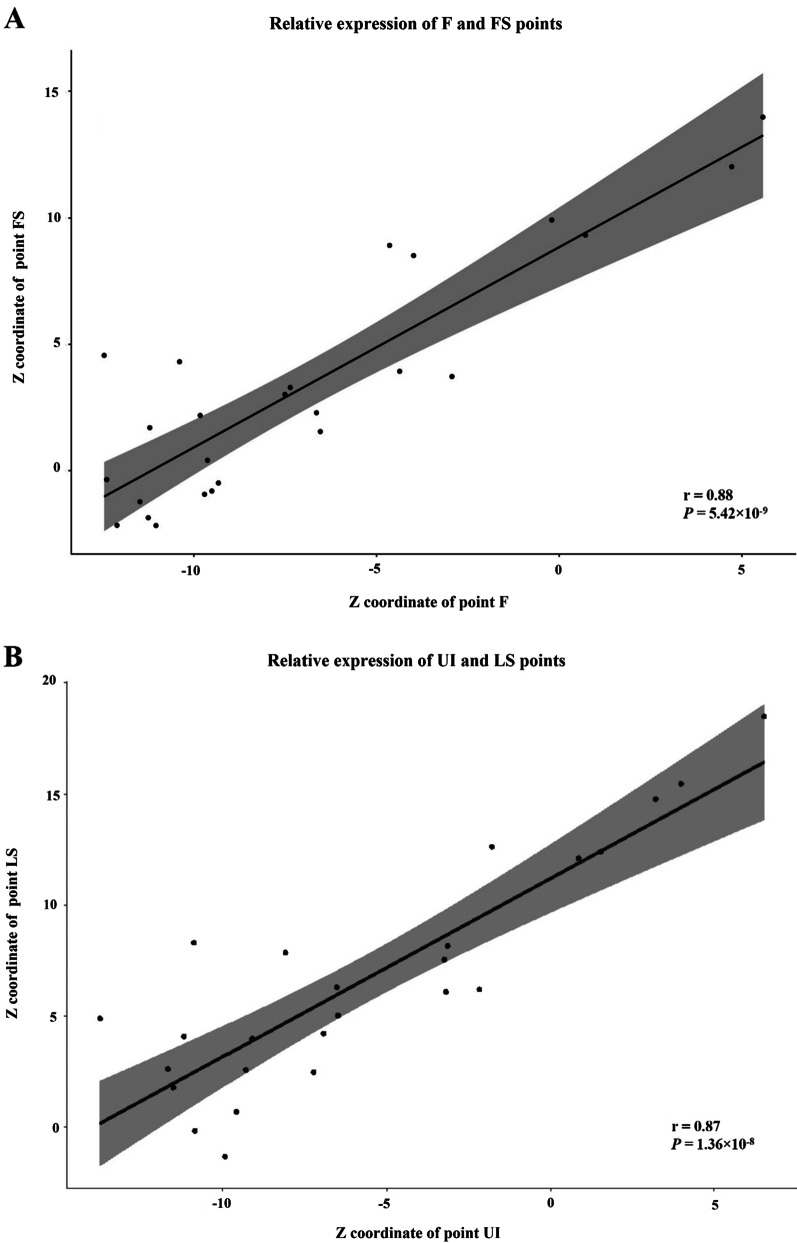
The Pearson correlations of clinically relevant corresponding prostheses and soft tissue changes in the Z-axis direction. **A** The relative expression of F and FS points. **B** The relative expression of UI and LS points

In the direction of the Z-axis, strong correlations were found not only between the movement of the FS and F (r = 0.88) but also between the movement of the LS and UI (r = 0.87) (Fig. 6). These two plots were produced using the package ggplot2 (Wicham, 2016). The linear regression equations are listed in Table 4: Z(FS) = 8.8389 + 0.7914 Z(F) and Z(LS) = 11.1936 + 0.8026 Z(UI).

**Table 4:** The clinically relevant corresponding prostheses and soft tissue changes in the Z-axis direction analyzed to derive the Pearson correlations and coefficients of determination

Soft tissue-to-prostheses relationship	Pearson correlation (r)	Coefficient of determination (R^2^ × 100)%	*P-*value for Pearson correlations
FS-F^a^	0.88	77.86	5.42 × 10^−9***^
LS-UI^b^	0.87	76.05	1.36 × 10^−8***^

^a^Linear regression equation: Z(FS) = 8.8389 + 0.7914 Z(F), n = 25, ***P ≤ 0.001

^b^Linear regression equation: Z(LS) = 11.1936 + 0.8026 Z(UI), n = 25, ***P ≤ 0.001

### Related influencing factors

**Fig. 7:**
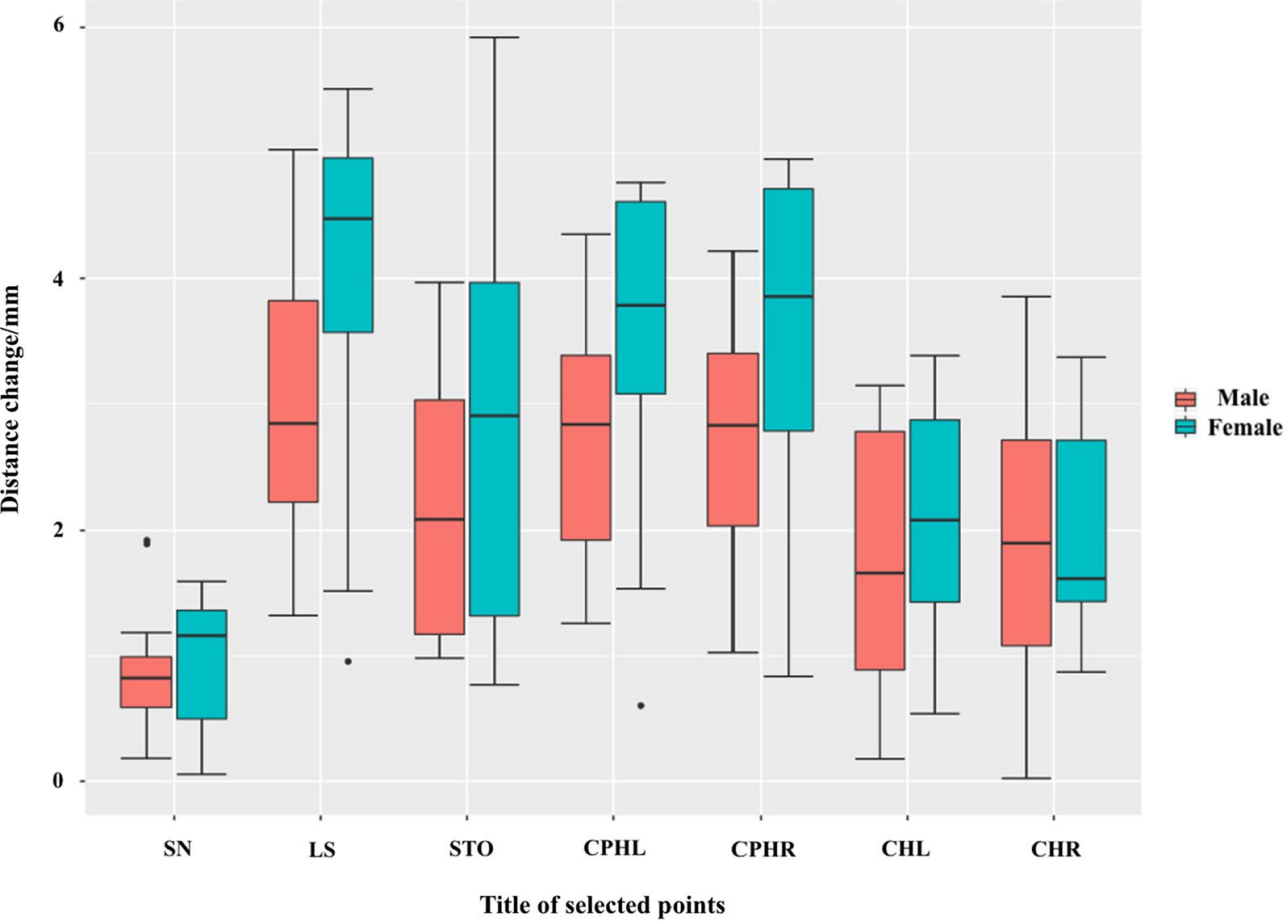
After the restoration, the landmarks’ variation on the Z-axis between males and females, *P < 0.05

**Fig. 8:**
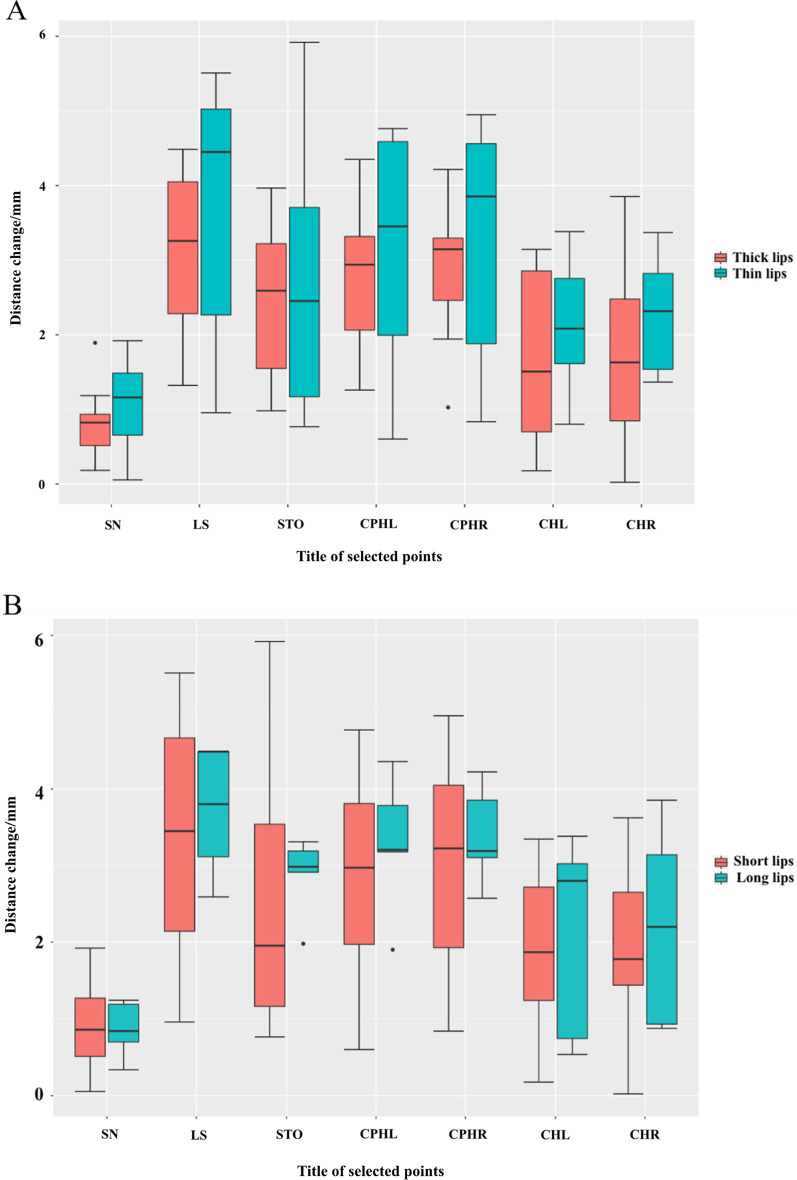
The landmarks’ shift on the Z-axis between **A** the thick and thin upper lips, **B** the short and long lips, *P < 0.05

The sagittal movement of the LS was different between the thin-lip group (3.77 ± 1.56 mm) and the thick-lip group (3.09 ± 1.14 mm) and the long-lip group (3.69 ± 0.84 mm) and the short-lip group (3.38 ± 1.50 mm). A similar tendency was also observed in other landmark movements (STO, CPHL, CPHR, CHL, CHR). The SN, LS, STO, CPHL, and CPHR of female patients’ lips moved much more forward than those of male patients (Fig. 7). Patients with a thinner ULT showed more lip support than patients with thicker ULT by a clinically insignificant amount, especially in the middle part of the upper lip. Moreover, patients with longer ULL showed more displacement than those with shorter ULL by an insignificant difference (Fig. 8).

## Discussion

The clinically acceptable accuracy of the 3D facial imaging system offers considerable improvement in stereophotogrammetry for facial capture and analysis. Khambay et al. [28] reported the system error of 3D facial imaging system was within 200 μm. The capture time of 3D facial imaging system is 50 ms and its accuracy is within 500 μm [31]. Although the mean global deviation between the multimodal image fusion model (220 ± 50 μm) and the laser-scanned model (170 ± 70 μm) showed the statistically significant difference, the mean difference of 500 μm is unlikely to have a clinical impact [32].

During the 3D data acquisition in this study, one of the three profiles was obtained using the visible frontal teeth in the intercuspal position. However, this profile was an intermediate image, which was only used to integrate the denture with the post-restoration profile. Therefore, the soft tissue changes in the intermediate image would not affect the measurement results of the soft tissue changes before and after the dental prostheses were worn.

After wearing the maxillary fixed dentures, the vertical distance between the LS and STO increased significantly, but the length of the upper lips (SN-LS) was reduced. This finding is consistent with the upward shift of the LS, CPHL, and CPHR points and the downward shift of the STO, CHL, and CHR points. The similar tendency of the upper lips to change after wearing the traditional removable complete dentures has been reported [5]. The reason why the soft tissue marks moved vertically and forwardly was that after denture wearing, the lower third of the facial height increased and the contact force and the tension of soft tissue changed, which influenced the shape and contour of the lips [33].

In 2017, a review about lip measurements in Asians claimed that mean female upper vermilion height was 9.09 mm, the mean male value was 10.20 mm, and the most attractive average value was 7.8 mm for the upper lips at the midline [34]. In this study, the average height of the upper lip in 25 edentulous patients before and after restoration were 5.02 mm and 6.43 mm, respectively. It can be observed that the shape of the upper lip was well restored following the maxillary implant-supported fixed denture.

In the horizontal direction, the CPHL, CPHR, CHL, and CHR showed a tendency to move to both sides. The moving distances of the CHL and CHR were slightly larger than those of the CPHL and CPHR. For maxillary edentulous patients, the resorption of the labial alveolar bone was more evident than that on the palatal side. The overall resorption caused the alveolar bone to move upward and inward, resulting in insufficient support of the upper lip. The maxillary denture restored the patients’ upper lip support and returned the CHR and CHL to the original position. The CPHL and CPHR moved away from each other because of the tension of the soft tissues. However, compared with the previous study on removable complete dentures, the degree of lip support associated with the implant-supported fixed denture was slightly less than that of the removable complete denture [9].

In general, soft tissues are anisotropic, viscoelastic, and nonlinear. However, it is usually assumed that they behave as linear, elastic, and isotropic materials to simplify the analysis [35]. The two pairs of landmarks (F-FS and UI-LS) have been proven to have strong positive movement correlations along the sagittal direction. The linear equations reflecting the relative spatial positions of the two points are Z(FS) = 8.8389 + 0.7914 Z(F) and Z(LS) = 11.1936 + 0.8026 Z(UI). The integration method and the equations could help doctors reconstruct the relative spatial position of the dentures and facial soft tissue in the same coordinate system and analyze or preliminarily predict the effect of the maxillary full-arch implant-supported fixed prostheses from an esthetic viewpoint.

This study selected the facial scanner and model scanner for the following reasons. First, artifacts of implants in cone-beam computed tomography (CBCT) images will affect the accuracy of later data registration. Second, several systematic reviews have revealed that the intraoral scanning technique does not appear to have the same accuracy as conventional impressions in the case of long-span restorations such as partial fixed prostheses with more than five elements or full-arch prostheses on natural teeth or implants [36, 37].

Women show more advancement of landmarks than men. However, a previous study on cephalograms and ULT of 240 Chinese adult patients has reported that there was statistical significance in ULT between men and women (*P* < 0.01) [30]. According to the Chinese Han national criteria for the thickness of the upper lip, 10 out of 11 women were assigned to the thin-lip group in our study, which means that it is probably the ULT, not the sex, that influenced the soft tissue changes.

After taking the ULT into consideration, although the amount of lip support of the fixed implant-supported denture is limited, patients with thinner lips also show more soft tissue changes. Kuhn et al. [6] analyzed 47 pairs of lateral cephalograms and found that lip retraction was less pronounced in patients with thicker lips than in those with thinner lips. After maxillary bone surgery, patients with thin lips experienced nearly three times more advancement of the lips than did patients with thick lips for the same amount of bone movement [38].

However, patients with longer upper lips showed a more evident change after denture fixation. The fixed implant-supported maxillary dentures make all the selected points of the upper lips move forward. The longer the upper lips, the more evident is the collapse of the soft tissue without the denture. To restore the normal lip support, all selected landmarks need to move forward much more. However, the influence of the length and thickness of the upper lips on the lip support was not statistically significant. Our results do not show a clear distinction between these groups and indicate that the reported difference may be difficult to identify in a homogenous sample not selected for lip thickness and lip length [6].

## Conclusions

The 3D integration method showed high accuracy and repeatability in reflecting the relative positions of implant-supported prostheses and facial soft tissue. After wearing the maxillary full-arch implant-supported fixed prostheses, the upper lip support was significantly increased, especially in the middle part of the lips. Except for the decrease in SN-LS, the lengths of CPHR-CPHL, CHR-CHL, and LS-STO increased significantly. The integration method and the linear regression equations between F and FS and UI and LS in the sagittal direction could aid dentists in reconstructing the real related position between the dentures and upper lips in the same coordinate system and preliminarily analyze the effect of the lip support reconstruction.

## Abbreviations

2D: Two dimensional
3D: Three dimensional
CBCT: Cone-beam computed tomography
LS: Labrale superius
CPHL: Crista philtri left
CPHR: Crista philtri right
SN: Subnasale
STO: Stomion
UI: Upper incisor
OCL: Left outer canthus
OCR: Right outer canthus
N: Nasion
F: The central top point of the acrylic flange border
CHL: Cheilion left
CHR: Cheilion right
FS: The intersection of the parallel line of UI-LS through point F and the surface of the soft tissue
ULT: Upper lip thickness
ULL: Upper lip length
SPSS: Statistical package for the social sciences
MAD: Mean absolute difference
TEM: Technical error of measurement

## Declarations

### Availability of data and materials

All data generated or analysed during this study are included in this published article.
